# Estimates of on-farm antimicrobial usage in egg production in the United States, 2016–2021

**DOI:** 10.3389/fvets.2023.1135377

**Published:** 2023-03-30

**Authors:** Randall S. Singer

**Affiliations:** ^1^Department of Veterinary and Biomedical Sciences, University of Minnesota, St. Paul, MN, United States; ^2^Mindwalk Consulting Group, LLC, Falcon Heights, MN, United States

**Keywords:** layer chickens, table eggs, antimicrobial use, antimicrobial stewardship, epidemiological monitoring

## Abstract

Very few data exist globally regarding the use of antimicrobials in the table egg industry. Antimicrobial use data from broiler chickens and turkeys cannot be used as a surrogate of layer chickens because of the fact that table eggs for human consumption are produced daily by laying hens. To avoid the possibility of antimicrobial residues in the eggs, there are very few antimicrobials approved for use in layers in the U.S. The objective of this study was to collect on-farm antimicrobial use data from the U.S. table egg industry and to have it be representative of the national layer flock. Participation was voluntary. Data were collected for the period 2016 through 2021 and are reported on a calendar year basis. Using production statistics from USDA:NASS as a denominator, the data supplied by participating companies accounted for 3,016,183,140 dozen eggs (~40% of national egg production) in 2016 and 3,556,743,270 dozen eggs (~45% of national egg production) in 2021. All of the replacement chicks placed on pullet farms during the study period were estimated to have received 0.2 mg/chick gentamicin at the hatchery. Most of the antimicrobial administration in U.S. egg production is *via* the feed. The ionophores monensin and salinomycin were used in the pullets, bacitracin was used in both pullets and layers (primarily for control of necrotic enteritis), and chlortetracycline was used primarily in layers for the treatment of *E. coli*-related disease. In the layers, between 0.10 and 0.19% of total hen-days were exposed to chlortetracycline. Only two water-soluble administrations were recorded during the entire study period, both involving lincomycin to pullet flocks for the treatment of necrotic enteritis. Overall, antimicrobial use in the U.S. layer industry was focused mainly on controlling necrotic enteritis in the pullets and treating *E. coli*-related disease in the laying hens.

## 1. Introduction

Antimicrobials are used in animal agriculture globally, but patterns of use vary considerably by country/region and by species. Much of the data being collected are sales data and provide no information about how these antimicrobials were actually used on the farm. Further, very few data exist globally regarding the use of antimicrobials in the table egg industry. Antimicrobial use (AMU) in layer chickens is very different from broiler chickens and turkeys. Table egg production is similar to milk production, where the product for human consumption is produced on a daily basis. Most antimicrobials that could be administered to laying hens have withdrawal periods that would prevent all eggs produced during this period from entering the food supply. For this reason, the antimicrobials approved for use in the U.S. differ between pullets and laying hens. In the U.S. very few antimicrobials are permitted for use in layers, and for those drugs that are allowed to be used in the laying hen, there is typically a non-zero-day withdrawal period. In the U.S. the only medically important antimicrobial that is allowed for use in laying hens and that has a zero-day withdrawal is chlortetracycline (CTC) administered in the feed.

Antimicrobial use in table egg laying chickens in the U.S. is influenced by the phase of production. In the U.S. there are three primary phases of commercial egg production: hatchery, pullet growing farms and egg laying farms. In the U.S., the majority of chicks purchased by egg companies are sourced from hatcheries that are owned and operated by day-old chick production companies. Consequently, data regarding antimicrobials administered to these chicks prior to arrival on the pullet farm would be held by the day-old chick production companies. Pullets are then raised on farms owned by the production company or contract growers until the birds are ~15–18 weeks of age. The birds are then transferred to egg laying farms which are often located on separate premises from the pullet farms. Hens will typically begin laying eggs around 20 weeks of age. The length of the productive life of a laying hen depends upon the number of egg production cycles utilized on the egg farm but typically ends when the hen is around 80–100 weeks of age.

The U.S. Food and Drug Administration (FDA) has made changes to antimicrobial policy in animal agriculture in recent years. In 2012, FDA released Guidance for Industry (GFI) #209 (1) which proposed eliminating the use of medically important antimicrobials for increased rate of weight gain and improved feed efficiency in food-producing animals. Further, the document proposed requiring veterinary oversight for the use of medically important antimicrobials administered in the feed or water of food-producing animals. FDA's GFI #152 (2) defines “medically important” antimicrobials (i.e., importance to human medical therapy), and Appendix A of that document provides a list and ranking of antimicrobials considered medically important in the U.S.; this list serves as the operating classification system for the data presented in this U.S.-based effort. In 2013, the FDA published GFI #213, which provided detail on the implementation of the key principles in GFI #209 (3), including guidance to drug sponsors on how to remove label claims relating to growth promotion/feed efficiency uses of medically important antimicrobials. Because neither the Animal Medicinal Drug Use Clarification Act nor the Veterinary Feed Directive (VFD) Rule allows extra-label use of animal drugs in the feed of food-producing animals in the U.S. (4), this voluntary removal of the label claims eliminated the use of medically important antimicrobials for production uses (growth promotion/feed efficiency) in the U.S. These changes were fully implemented by January 2017.

In the U.S., summary reports are issued annually by the FDA of the amounts of antimicrobials sold or distributed for use in food-producing animals (5). The challenge with sales data, though, is that they lack the granularity to determine the reason for antimicrobial administration and other details such as age at administration, dose, route and duration. Further, the sales data for the U.S., which have been stratified by species-specific estimates since 2016, are best guesses by the drug manufacturers regarding the species in which the product would ultimately be used. These species-specific estimates include “chickens” which would include both broiler chickens and layers and therefore these data provide no insight into how antimicrobials are used in table egg production in the U.S. The objective of this study was to collect on-farm AMU data from the U.S. table egg industry and to have it be representative of the national layer flock and national egg production. Because of the changes made to antimicrobial regulations in the U.S. in January 2017, data were collected for the years 2016 through 2021, thus straddling the full implementation of U.S. Food and Drug Administration (FDA) Guidance for Industry (GFI) #213 and the changes to the VFD rule.

## 2. Materials and methods

### 2.1. Enrollment

The goal of this effort was to enroll companies that produce table eggs in the U.S. A list of the largest egg production companies in the U.S. is published annually by WATT Poultry USA (https://www.wattglobalmedia.com/publications/poultry-usa/). The published list in January 2022 included the largest 67 companies in the U.S., ranked according to the number of hens in lay at the end of 2021. The list includes all types of production and management, for example conventional and organic egg production. According to national estimates from the USDA National Agricultural Statistics Service (USDA:NASS), ~7,964,226,500 dozen eggs were produced in 2021 in the U.S., with an average hen inventory of 323,008,250 (6). Although the WATT list does not represent every table egg producer in the country, the companies on the list account for the vast majority of eggs produced in the U.S. each year. Companies on the list represent a diversity of production types, including conventional caged, cage-free, enriched colony, free-range and organic. Many of the companies produce eggs in more than one of these production systems. The list was used as a list frame to identify companies that should be contacted to participate in the study and was not used as a source of data included in this project.

Companies on the WATT list were contacted with the assistance of the United States Poultry & Egg Association (USPOULTRY), United Egg Producers, American Association of Avian Pathologists, and Association of Veterinarians in Egg Production. The companies that agreed to participate were informed that they would need to provide data from 2016 through 2021 regarding production parameters, antimicrobials used at the hatchery, and antimicrobials used in the feed and water of pullets and laying hens. Companies were also asked to provide data regarding the indication for use. Participation was voluntary. All companies were guaranteed data confidentiality and that only industrywide aggregated data would be released publicly.

### 2.2. Antimicrobial use

The data collected from the participating layer companies were aggregated into calendar year totals. Because of the arrangement of the U.S. table egg industry wherein most of the chicks are hatched by day-old chick production companies, data regarding antimicrobial administration in the hatchery were often retrieved from the day-old chick production companies with the permission of the participating production company.

The data were provided by the participating companies in a variety of formats, thereby making a single, standardized approach to data collection practically impossible. Regardless of format, all companies submitted data regarding annual number of pullets placed, annual number of eggs produced, and average number of hens in lay. Data for average number of hens in lay is typically recorded by the companies on a weekly basis, and the average of these weekly estimates over the calendar year was used as the average annual number of hens in lay for that company. The use of ionophores in the pullets (no ionophore use is approved for hens in lay) and other not-medically important (NMI) antimicrobials do not require a prescription from a veterinarian, and therefore, these data were obtained from the feed mills, nutritionists or the veterinarians. Since January 2017, usage of medically important antimicrobials in the feed or water requires veterinary oversight in the U.S., and thus these data were often collected directly from veterinary records kept within the company. The medically important antimicrobial administrations could also be split into treatment of the pullets or the layers. For the NMI data, this division was not always possible.

Data from each company were first validated in individual spreadsheets (Microsoft Excel, Microsoft Corporation, Redmond, WA, USA). Errors were often identified during this process, and any data points that appeared to be erroneous were reviewed and confirmed with the submitting company. A relational database was created (Microsoft Access, Microsoft Corporation, Redmond, WA, USA), and records were then imported into this database. Customized Visual Basic scripts further assisted with error checking and data validation. Once in the same format, data from all companies were then aggregated.

For each antimicrobial, the total kilograms of active ingredient used in each calendar year was calculated. Several denominators were generated for the numerator data. First, the annual average inventory of hens in lay and the total number of dozens of eggs produced during the year were used to standardize the annual antimicrobial numerators. Second, total annual hen-days was calculated by multiplying the average number of hens in lay in the calendar year by 365. This denominator could then be compared to the numerator of total number of hen-days exposed to a medically important antimicrobial. These hen-day calculations ignore the amount of antimicrobial used in the pullet phase. All antimicrobial use data collected during this study are included in the Supplementary material.

## 3. Results

### 3.1. Enrollment

The companies that voluntarily submitted data for this project represented a sizeable fraction of U.S. egg production during each year of the study. The companies that participated encompassed all types of egg production, including caged and cage-free management and conventional and organic production systems. Individual companies can utilize multiple production types, and the data that were provided do not breakdown the amount of production by system type. The data that were submitted in 2016 covered an average hen inventory of 125,607,224 hens and annual production of 3,016,183,140 dozen eggs, which is more than 40% of annual production in the U.S. (Table 1). In 2021, submitted data covered an average hen inventory of 148,128,896 hens and 3,556,743,270 dozen eggs produced, representing ~45% of annual production in the U.S. (Table 1). All denominator data collected during this study are included in the Supplementary material.

**Table 1:** Layer production data included in the antimicrobial datasets submitted by participating companies for each year of the study.

	**2016**	**2017**	**2018**	**2019**	**2020**	**2021**
**Study production data**						
Number chicks placed	94,733,656	94,264,336	97,399,815	98,563,669	101,308,162	101,942,713
Average hen inventory	125,607,224	131,367,570	136,981,763	140,748,388	146,007,726	148,128,896
Eggs produced (Dozen)	3,016,183,140	3,178,037,700	3,171,355,740	3,383,631,600	3,579,474,030	3,556,743,270
**USDA:NASS statistics**						
Average hen inventory	311,999,500	321,819,833	333,604,250	336,856,333	326,530,667	323,008,250
Eggs produced (Dozen)	7,441,160,700	7,799,967,900	8,012,785,800	8,254,557,400	8,089,332,200	7,964,226,500
**Percentage of U.S. egg production**						
Average hen inventory	40.3%	40.8%	41.1%	41.8%	44.7%	45.9%
Eggs produced (Dozen)	40.5%	40.7%	39.6%	41.0%	44.2%	44.7%

Annual U.S. egg production statistics are taken from USDA:NASS (6). The percentage of annual U.S. egg production calculation is based on the study production data (numerator) and USDA:NASS data (denominator).

### 3.2. Antimicrobial use

**Figure 1:**
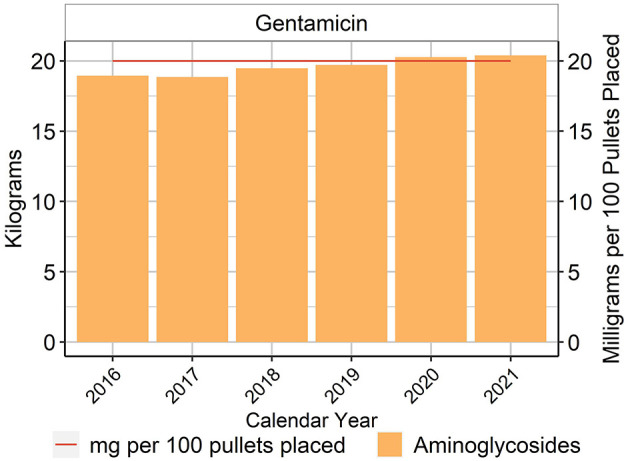
Gentamicin used in layer hatcheries, 2016–2021. Total kilograms are shown by the bars (left Y-axis) and total milligrams/100 birds placed are shown by the line (right Y-axis).

Data regarding the use of antimicrobials administered to the chicks at the hatchery were obtained primarily from the day-old chick production companies that source the majority of replacement chicks to the production companies. For the dataset included in this report, 100% of placed chicks received gentamicin at the hatchery at a standard dose of 0.2 mg/chick. The label of gentamicin sulfate for use in chickens states that it is recommended for the prevention of early mortality in day-old chickens associated with *E. coli, Salmonella* Typhimurium and *Pseudomonas aeruginosa* susceptible to gentamicin sulfate. The annual estimated gentamicin amount used in the chicks included in this study ranged between 18.9 and 20.4 kg (Figure 1), depending on the year. Because all chicks in the dataset received gentamicin, the amount of gentamicin per 100 pullets placed was fixed at 20 mg for all years (Figure 1).

**Figure 2:**
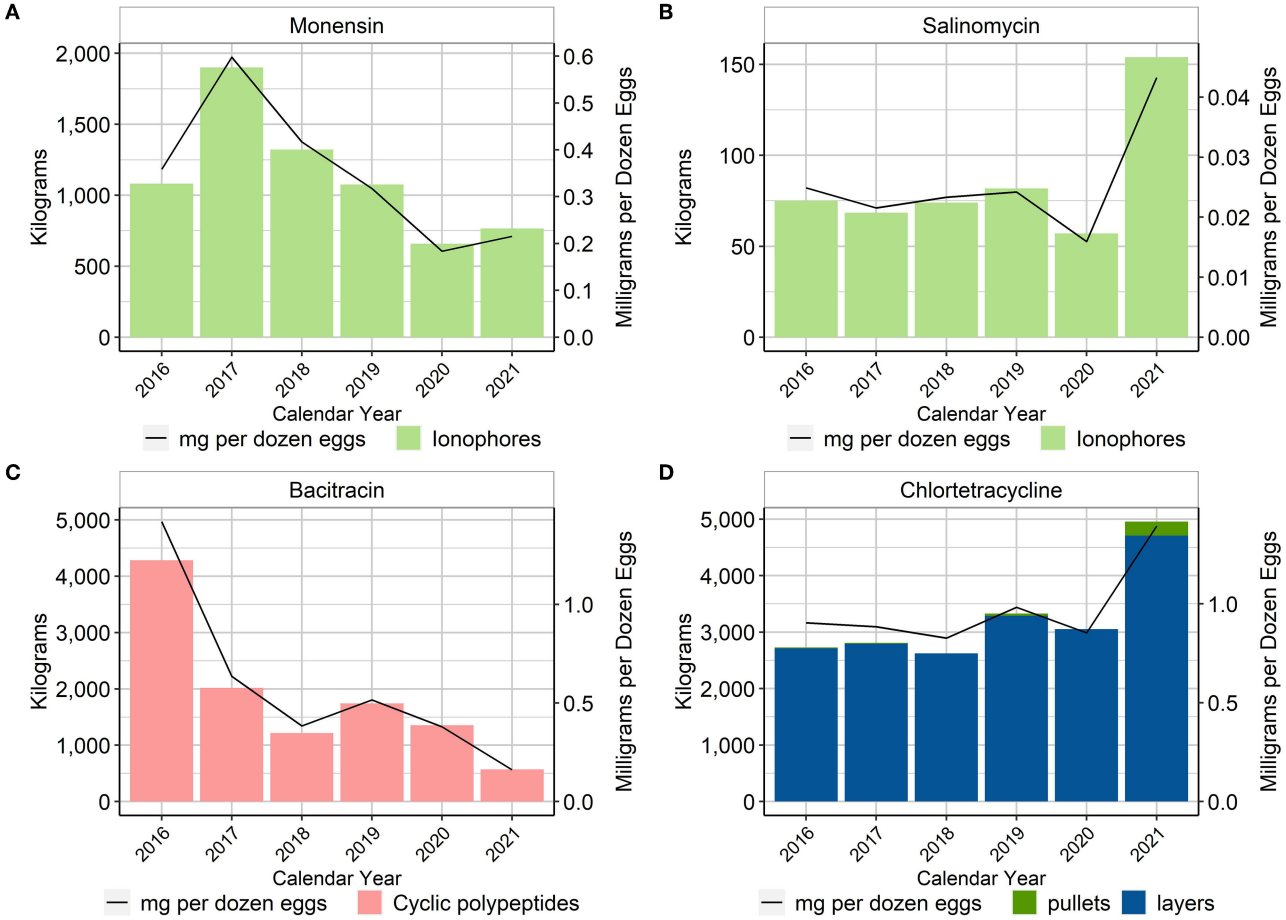
Monensin **(A)**, salinomycin **(B)**, bacitracin **(C)**, and chlortetracycline **(D)** used in layer feed, 2016–2021. Total kilograms are shown by the bars (left Y-axis) and total mg/dozen eggs produced are shown by the line (right Y-axis). Total kilograms of chlortetracycline are separated into pullet and laying hen use.

Although ionophores are considered anticoccidials and not antimicrobials in many countries, for example in the European Union where they are considered coccidiostats (7), they are classified as NMI antimicrobial drugs in the U.S. There are currently two ionophores approved for use in the U.S. layer industry: monensin and salinomycin (8). These compounds are approved for use in the pullets, but neither is allowed for use in the laying hen. Both monensin and salinomycin were used in the pullet phase in all years of the project. When using the metric of total mg/dozen eggs produced, there was an approximate 40% reduction of monensin use while salinomycin use remained fairly stable over the period 2016–2020 and then more than doubled in 2021 (Figure 2).

Only two in-feed antimicrobials, other than the ionophores, had reported use in U.S. layer production. In feed bacitracin, an NMI antimicrobial in the U.S., was used in both pullets and layers (Figure 2). From 2016 to 2017, there was an approximate 55% reduction in the use of bacitracin when using the metric of total mg/dozen eggs produced; the use of bacitracin then stabilized from 2017 to 2021. Because bacitracin is an NMI antimicrobial, a VFD is not required. Consequently, data concerning flock specifics were not always available and thus the specific administrations could not always be separated into pullet vs. layer. The maximum inclusion rate of bacitracin in layers in the U.S. is 25 g/ton of feed for the treatment and control of necrotic enteritis, whereas in pullets, bacitracin can be used at an inclusion rate of 100–200 g/ton of feed for the prevention and control of necrotic enteritis.

The second in-feed antimicrobial used in U.S. layer production was chlortetracycline (CTC). Most of this use was in the layers for the treatment of disease related to *E. coli*. The two main uses of CTC in the layers were (1) for the control of chronic respiratory disease (CRD) and air sac infection caused by *E. coli* at an inclusion rate of 400 g/ton of feed for 10–14 days, and (2) for the reduction of mortality due to *E. coli* infections susceptible to chlortetracycline at an inclusion rate of 500 g/ton of feed for 5 days. As stated previously, because these are in-feed administrations in the U.S., it is illegal to use this antimicrobial in an extra-label manner (4).

Figure 2 shows the total amount of CTC used in pullets and layers over the reporting period as well as CTC used in pullets and layers expressed with the metric mg/dozen eggs produced. Because the pullet contribution was minimal, it can be difficult to visualize in the figure, but there was usage in the pullets in 2016, 2017, 2019, and 2021 (Supplementary material). The percentage of total annual hen-days exposed to CTC was also calculated, using the total number of CTC treatment days in hens (numerator) divided by the total number of hen-days in the calendar year. Between 0.10 and 0.19% of total hen-days were exposed to CTC.

**Figure 3:**
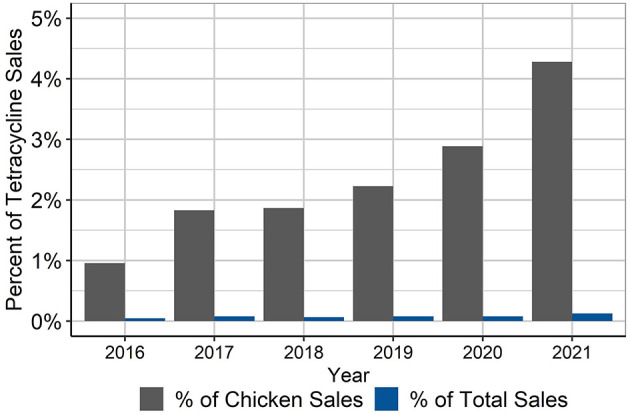
Percentage of tetracycline sales in U.S. animal agriculture that is represented in the CTC use in layers in this dataset, 2016–2021 (5). Estimates are shown for percentage of tetracyclines sold for use in chickens and for all food-producing animals.

Finally, the percentage of total tetracycline sales in the U.S. that are represented by the CTC volumes used in this study period was calculated, both as a percentage of the estimated chicken sales and the percentage of total estimated tetracycline sales (Figure 3). Given that the production in this dataset represents about 45% of annual production, it is unknown how much additional CTC was used in the companies that were not included in this study. The CTC used by the companies in this study represented ~4.3% of estimated chicken tetracycline sales and ~0.13% of total tetracycline sales in 2021, and much less in the years 2016–2020. Without sales data specific to the layer industry, it would be impossible to know how much of the tetracycline sales were for broilers vs. layers because the sales data cannot stratify the species-specific estimates beyond “chickens;” the species-specific estimates are also only best guesses by the manufacturers.

Water-soluble antimicrobials are used rarely in table egg production. Over the study period, there were only two reported water-soluble antimicrobial administrations in the participating companies, and both of these administrations were in the pullet phase and were prescribed for the treatment of necrotic enteritis. One pullet flock in 2017 of ~25,000 birds received lincomycin for 7 days, and in 2018 a second pullet flock of ~38,500 birds received lincomycin for 10 days.

## 4. Discussion

This report describes the first industrywide effort to capture antimicrobial use data in the U.S. table egg industry. The study period was 2016 to 2021. Thanks to the voluntary participation of many of the major table egg companies of the U.S., participation rates were high; ~45% of 2021 U.S. egg production as reported by USDA:NASS was included in this analysis (Table 1). Overall, very little antimicrobial is used in the U.S. layer industry. The main antimicrobials used in layer production are the NMI antimicrobial bacitracin (used as bacitracin methylene disalicylate, BMD) and the MI antimicrobial CTC. Both of these in-feed administrations have a zero-day withdrawal for eggs. The use of gentamicin in the hatchery and CTC in the feed of pullets and layers were the only two MI antimicrobials used in this dataset covering 6 years of production. The data in this study straddle the January 2017 date when changes to the in-feed and water-soluble uses of antimicrobials in U.S. animal agriculture were fully implemented. Beginning in 2017, CTC use required veterinarian approval through a VFD. BMD and the ionophores, however, are NMI antimicrobials in the U.S. and therefore do not require veterinary oversight or a VFD, although many companies assign the responsibility of all antimicrobial administrations to the veterinary group.

Necrotic enteritis is primarily caused by *Clostridium perfringens* and remains an important cause of morbidity and mortality in the pullet phase of production. The disease is characterized by sudden onset, high mortality, and necrosis of the mucous membrane of the small intestine (9) and is sometimes exacerbated by co-infection with coccidia. In the layer phase of production, disease related to *E. coli* is the main reason for CTC use. However, there are other disease challenges that the pullets and layers face, including infectious coryza, which is an acute respiratory disease of chickens caused by the bacterium *Avibacterium paragallinarum* (10).

For these rapidly progressing diseases, such as infectious coryza and some *E. coli* infections, some veterinarians in this study expressed a concern that there are no water-soluble antimicrobials approved in the U.S. that have a zero-day withdrawal. In Australia, for example, there are zero-day withdrawal approvals of water-soluble CTC, amoxicillin, and lincomycin-spectinomycin combination product (11). Without an approved water-soluble administration in the U.S., the use of water-soluble CTC would have a minimum one-day withdrawal and would result in the destruction of many eggs, leading companies to select the approved in-feed CTC administration. Having approved water-soluble options is desirable, as sick birds are likely to continue drinking but may stop eating (12). Further, water-soluble antimicrobials can be easier to administer to a single barn instead of having to mix a specific medicated ration for a small number of birds.

There are limited published data regarding antimicrobial use in table egg production, but the data that are available make it clear that usage estimates cannot be compared across countries. The British Egg Industry Council (BEIC) is one of the only national AMU datasets that includes data on the table egg industry (13). For 2021, the reported data represented ~90% of the industry, where the national flock is estimated to include 43 million hens. The report estimated that 0.47% and 0.33% of total hen-days were exposed to an antimicrobial in 2020 and 2021, respectively (13). While the data in this U.S. study had a lower estimate of exposure frequency in layers (ranging from 0.10 to 0.19%), the U.S. estimates do not include the use of BMD in the layers due to lack of granular data for this antimicrobial. The UK data reports that the main MI antimicrobial class used was the tetracyclines, similar to the U.S. data. However, the UK report also states that other classes used in the laying hens included pleuromutilins, macrolides, penicillins, aminoglycosides, fluoroquinolones, polymyxins, sulfonamides, lincosamides and combination products (13). Some of these compounds are illegal to use in U.S. layers, and none of these classes was used in U.S. layers, in part because of the lack of water-soluble options in the U.S. with zero-day withdrawal.

Reports of AMU in the Netherlands (14) focus on the use of colistin in layers; this antimicrobial has never been approved for use in animal agriculture in the U.S. Other classes used in layer production in the Netherlands included polymixins, macrolides/lincosamides, penicillins, pleuromutilins, and a small amount of tetracyclines. Many of these classes were also used in layer pullets. The report from the Netherlands (14) does not detail the amounts of these classes used or the diseases targeted but instead reports the frequency of farms that used each antimicrobial class. In Denmark, national data are reported by DANMAP, but the report only includes sales data by species, which separates layers from broiler and turkeys (15). In 2021, penicillins and tetracyclines were the two primary antimicrobials sold for use, reported on a kg basis. These sales data provide no distinction between pullets and layers and also provide no information regarding diseases targeted by treatment. The DANMAP report includes usage data, but the data for poultry do not separate the individual poultry commodities. Finally, there are individual studies reporting antimicrobial use in layers in other countries, but these studies are not intended to be representative of national production. In Nigeria, there was reported use of gentamicin, tetracycline, enrofloxacin, ciprofloxacin, penicillin, streptomycin, chloramphenicol, erythromycin, and others in laying hens (16). According to this study, much of the use was prophylactic. In a study from Bangladesh, antimicrobials such as ciprofloxacin, amoxicillin, tetracyclines, macrolides, and others were used in layer production (17). Finally, a survey conducted in Brazil of veterinarians working with layers reported that fluoroquinolones and macrolides were two of the most common antimicrobials used (18). With different countries having different antimicrobial classes approved for use in layer chickens, and very few of the datasets including on-farm usage, it would seem ill-advised to make comparisons of AMU in the table egg industry across countries.

In this study, 100% of the chicks placed on pullet farms likely received gentamicin at a dose of 0.2 mg/chick at the hatchery. This is due, in large part, to the unique contractual arrangements between the day-old chick production companies who own the hatcheries and the commercial operations who source their replacement pullets from the day-old chick production companies. This administration of gentamicin to the chicks includes those that will be raised on operations that will eventually sell their eggs as organic. Whereas, the organic rule in the U.S. prohibits all uses of antimicrobials in animals or animal products that will be marketed as organic (19), even if the animal is sick, the organic designation for poultry starts after 72 h (second day of life on the farm that is being managed as organic). Specifically, the organic rule in the U.S. states “Poultry or edible poultry products must be from poultry that has been under continuous organic management beginning no later than the second day of life” (19). This rule, which prohibits the treatment of sick animals with antimicrobials and thus might seem contrary to the ideals of good animal welfare, is different than the organic rule in other locations, such as in Europe, where the rule states that “antibiotics may be used under the responsibility of a veterinarian” (20).

There were limitations to this first effort to collect antimicrobial use data from U.S. layer production. First, the effort targeted the major table egg companies that are included on the annual WATT Poultry USA list; although this list might miss some of the small producers, the majority of U.S. production is included in this list. Future efforts will seek to enroll more companies on this list and then expand to the small producers not included on the list. Second, the companies that were enrolled included different production types: conventional caged, cage-free, enriched colony, free-range and organic. Many of the companies produce eggs in more than one of these production systems. A limitation of the collected data is the inability to stratify the usage information by production type, as companies tend to use more than one production type. Antimicrobial usage information is only for flocks that were exposed to an antimicrobial, whereas the denominator information is for the entire company within a given year. Third, granularity in the data regarding the NMI antimicrobials (ionophores and bacitracin) was not as good as the data for CTC administrations. This is due, in large part, to the requirement that a VFD be completed for all in-feed administrations of MI antimicrobials. Going forward, this project will work with participating companies to improve the recordkeeping of all antimicrobial uses.

Overall antimicrobial use in the U.S. layer industry appears to be minimal, focused mainly on controlling necrotic enteritis in the pullets and treating *E. coli*-related disease in the laying hens. The only antimicrobials used in layer production were CTC, bacitracin, and two ionophores. Gentamicin was used in the hatchery. Regardless, there are still opportunities to improve antimicrobial stewardship, for example, by improving recordkeeping, ensuring that all uses of CTC are necessary and effective, and by helping veterinarians in the U.S. table egg industry obtain access to effective water-soluble therapies for key layer chicken diseases.

## Data availability statement

The original contributions presented in the study are included in the article/Supplementary material, further inquiries can be directed to the corresponding author.

## Author contributions

RS recruited the participants, coordinated data collection, conducted data analysis, and wrote the manuscript.

## Supplementary material

Supplementary material 1 Supporting Information LayerData2022.xlsx.
